# Epstein-Barr virus DNA modulates regulatory T-cell programming in addition to enhancing interleukin-17A production via Toll-like receptor 9

**DOI:** 10.1371/journal.pone.0200546

**Published:** 2018-07-11

**Authors:** Noor Salloum, Hadi M. Hussein, Rana Jammaz, Sara Jiche, Imad W. Uthman, Alexander M. Abdelnoor, Elias A. Rahal

**Affiliations:** 1 Department of Experimental Pathology, Immunology and Microbiology, American University of Beirut, Beirut, Lebanon; 2 Department of Internal Medicine, American University of Beirut Medical Center, Beirut, Lebanon; University of British Columbia, CANADA

## Abstract

Infection with the Epstein-Barr virus (EBV) has been associated with several autoimmune diseases including rheumatoid arthritis (RA). We have previously reported that DNA from this virus enhances production of the pro-autoimmune interleukin 17A (IL-17A) in mice. In this study we assessed the effect of EBV DNA on regulatory T cell programming and examined whether it mediated its effects via Toll-like receptor 9 (TLR9) in mice; moreover, we evaluated whether EBV DNA in humans had similar effects to those seen in mice. For this purpose, we assessed the linearity of the correlation between EBV DNA and IL-17A levels in RA subjects and matched controls. A modulatory effect for the viral DNA was observed for regulatory T cell markers with an inhibitory effect observed for CTLA4 expression in the EBV DNA-treated mice. To examine whether TLR9 mediated the detection of EBV DNA and enhancement of IL-17A production, mouse peripheral blood mononuclear cells were treated with the DNA in the presence or absence of the TLR9 inhibitor ODN 2088. Subsequently, IL-17A production from these cells was assessed. Treatment with the TLR9 inhibitor resulted in a significant decrease in IL-17A production indicating that TLR9 is involved in this pathway. In human subjects, examining the linearity of the correlation between EBV DNA and IL-17A levels in RA subjects showed a propensity for linearity that was not observed in controls. Our data thus indicates that EBV DNA itself acts as a modulator of the Th17 compartment as well as that of regulatory T cell mechanisms. The involvement of TLR9 in the EBV DNA-triggered induction of IL-17A suggests therapeutic targeting of this endosomal receptor in EBV positive subjects with an autoimmune flare-up or possibly for prophylactic purposes.

## Introduction

The Epstein-Barr virus (EBV), also known as *Human herpes virus 4*, is a member of the herpes family of viruses; its genome consists of a linear double-stranded DNA. More than 90% of adults are infected by this virus, hence EBV is highly prevalent worldwide [1]. As with other members of the *Herpesviridae*, EBV establishes latency in the infected host with potential recurrence of viral replication and shedding. EBV infection occurs through person-to-person transmission [2]. The majority of EBV infections are transmitted orally via saliva, but it could also be transmitted sexually and through blood transfusions. A primary infection with EBV during childhood is mostly asymptomatic. However, if the acquisition of EBV infection occurs in adolescence, which typically happens in developed countries [3], it usually causes infectious mononucleosis (IM).

EBV is associated with several types of malignancies [4] such as Burkitt's lymphoma, Hodgkin’s lymphoma, post-transplant lymphoproliferative disorders, and nasopharyngeal carcinoma. EBV is also associated with several autoimmune diseases such as rheumatoid arthritis (RA), systemic lupus erythematosus (SLE), and multiple sclerosis (MS) [5]. Studies have shown that EBV is the most common potential infectious trigger for RA [6]. Moreover, various studies have indicated higher viral loads, increased levels of anti-EBV antibodies, in addition to a compromised cell-mediated control of EBV infected cells in patients with autoimmune diseases compared to controls [5]. Multiple studies have also demonstrated possible epitope similarities between particular EBV-peptides, such as EBV Nuclear Antigen 1 (EBNA-1), a viral component of EBV, and myelin basic protein (MBP), the autoantigen in MS [7, 8]. This may underlie molecular mimicry and hence an autoimmune reaction [9–11]. In addition, antibodies directed against EBNA-1 have been shown to cross react with double stranded DNA, the autoantigen in SLE [12].

We have previously reported an increase in the levels of IL-17A, a pro-inflammatory cytokine, in mice injected with EBV DNA [13]. IL-17A plays a role in host defenses against particular bacterial and fungal pathogens; it results in the recruitment of neutrophils and macrophages to the site of infection and triggers the secretion of various pro-inflammatory mediators from multiple cell types. However, a pathologic role for this cytokine has also been reported as it has been associated with various autoimmune diseases. Numerous studies have demonstrated the involvement of T-helper 17 (Th17) cells, the primary producers of IL-17A, in autoimmunity [14–16]. For example, T cells in the synovial fluid of RA patients were shown to produce high amounts of IL-17A [17]. The involvement of Th17 cells in autoimmune diseases appears to be via promoting inflammation through the production of IL-17A in addition to IL-21 and IL-22. These cytokines then stimulate different types of cells to secrete pro-inflammatory molecules such as IL-1, IL-6 and TNF-α among others [18].

Studies have shown that particular CpG motifs and DNA sequences present in the genomes of bacteria and some viruses, such as *Herpes simplex virus 1* (HSV-1) and EBV, can activate the immune system promoting Th1-like responses [19–21]. Nascent EBV DNA contains unmethylated CpG dinucleotides [22] which are immunostimulatory components that appear to mediate responses via activation of Toll-like Receptor 9 (TLR9) [21]. To determine the mechanism through which EBV DNA triggers IL-17A production in mice, we examined whether TLR9 is involved. Moreover, we assessed whether the effects of EBV DNA extend to affect regulatory T cells (Tregs). We additionally examined whether humans are similarly affected by EBV DNA via examining the linearity of the relationship between IL-17A and EBV DNA levels in RA subjects and controls.

## Materials and methods

### Mice

Female BALB/c mice, 4–6 weeks of age, were obtained from the Animal Care Facility at the American University of Beirut (AUB) and were treated according to the guidelines of the Institutional Animal Care and Use Committee (IACUC) at AUB.

### Assessing expression of Th17 and Treg markers in EBV DNA-treated mice

To assess the effect of EBV DNA on the expression of Th17 and Treg markers, 27 female BALB/c mice were used. These mice were divided into 3 groups, each including 9 mice. One group received an injection of 144x10^3^ copies of EBV DNA (Advanced Biotechnologies, Columbia, MD), another group received 28.3 pg (equivalent to the weight of 144x10^3^ copies of EBV DNA) of *Staphylococcus epidermidis* DNA as a non-viral DNA control while a third group received sterile water, the vehicle. We used 144x10^3^ copies of DNA based on our previous studies showing this copy number to induce the highest levels of IL-17A in BALB/c mice [13]. All injections consisted of 100 μl and were performed intraperitoneally. Three mice were sacrificed per group on days 3, 6, and 9 post-injection. The spleens were collected and splenic cells were isolated for RNA extraction, using Qiazol. cDNA synthesis was performed using the extracted RNA and employing the QuantiTect® Reverse Transcription Kit (QIAGEN, Hilden, Germany) according to the manufacturer’s instructions. Real-time PCR was then carried out on these samples to detect the relative gene expression of RAR-related orphan receptor gamma (thymus) (RORγT), IL-17A, CD4 in addition to Forkhead box P3 (FOXP3) and cytotoxic T-lymphocyte antigen-4 (CTLA4). Primers were obtained from Macrogen (Seoul, South Korea). Primers used to assess the expression of RORγT and CTLA4 were designed using the NCBI primer designing tool whereas previously published primers were used to assess the expression of IL-17A [23], CD4 [24], FOXP3 [25] and β-actin [26]. Primers, annealing temperatures, and product lengths are detailed in Table 1. Relative expressions per sample normalized to that of the water-injected calibrator group on day 3 were then calculated using the ΔΔCT method [27]. Three replicates of the experiment were used for expression analysis.

**Table 1:** Primers used for real-time PCR.

Gene	Primers	Annealing temperature	Product length (bp)
RORγT	F: 5'-GACTTTCCCTCTGGCACACA-3'	56°C	135
	R: 5'-ATCCGGTCCTCTGCTTCTCT-3'		
IL-17A	F: 5'-TTAAGGTTCTCTCCTCTGAA-3'	56°C	104
	R: 5'-TAGGGAGCTAAATTATCCAA-3'		
CD4	F: 5’-GAGAGTCAGCGGAGTTCTC-3’	55°C	329
	R: 5’-CTCACAGGTCAAAGTATTGTTG-3’		
FOXP3	F: 5'-TGGGTGTCAGGAGCCCACCAG-3'	60.3°C	91
	R: 5'-AGGGCCACAGCATGGGTCTGT-3'		
CTLA4	F: 5'-GCCAGTGGTTCCAAAGGTTG-3'	60.9°C	133
	R: 5'-CACTGTGGGACGACACTGAT-3'		
β -actin	F: 5'-GGCATTGTTACCAACTGGGACGAC-3’	58.6°C	218
	R: 5'-CCAGAGGCATACAGGGACAGCACAG-3’		

### Examining the role of TLR9 in EBV DNA-triggered IL-17A production from mouse PBMCs

To examine the role of TLR9 in the EBV DNA-triggered IL-17A production, mouse PBMCs from female BALB/c mice, 4–6 weeks of age were separated and subsequently used in culture. The PBMCs were cultured in 96-well plates with each well contained 0.25x10^6^ cells in a total volume of 250μl of RPMI 1640 culture medium (Sigma-Aldrich Chemie GmbH, Munich, Germany) supplemented with 10% FBS (Sigma-Aldrich Chemie GmbH, Munich, Germany) and 1% penicillin-streptomycin (Lonza, Basel, Switzerland). Cells were cultured with either EBV DNA (9x10^3^ copies) alone, the TLR9 inhibitor ODN 2088 (Integrated DNA Technologies, Leuven, Belgium) (1.4μM) alone, both EBV DNA and TLR9 inhibitor, or *Staphylococcus epidermidis* DNA (1.7pg, equivalent to weight of 9x10^3^ copies of EBV DNA) as a non-viral DNA control. We employed 9x10^3^ copies of EBV DNA since this copy number induced maximal production of IL-17A *ex vivo* under the assessed conditions. In addition, untreated cells grown in culture medium were examined as well. The above conditions were replicated in quadruplets. Cells were incubated for 24 hours at 37° C with 5% CO_2_. Supernatants were then collected and assessed by ELISA (Abcam, Cambridge, UK) for the levels of IL-17A. ELISA assessments were performed in duplicates.

### Assessing the correlation between EBV DNA copy numbers and IL-17A levels in RA patients and controls

A total of 24 RA patients [age range 25–72 years, 3 males and 21 females; non-pregnant and fulfilling the American College of Rheumatology (ACR) classification criteria for RA] and 24 non-RA control subjects (sex and age-matched with patients, non-pregnant and with no immediate family history of autoimmune diseases), were recruited. Recruitment was restricted to subjects who did not have a flare-up and that were not on any immunosuppressive or antiviral medications. Institutional Research Board (IRB) approval was obtained prior to sample collection, and consent forms were signed by the participants. Blood samples were collected and serum was separated and later used for detection of IL-17 levels by ELISA. IL-17A levels were measured in human subject sera using human cytokine specific ELISA kits (Abcam, Cambridge, UK), according to the manufacturer’s recommendations. Assessments were performed in duplicates. Subject blood was also used to separate PBMCs using Ficoll-isopaque and were then subjected to DNA extraction using phenol followed by precipitation with ethanol. To determine the EBV DNA load in human samples, Quantitative Real-Time PCR (qPCR) was performed, in triplicates, to quantitate the level of EBV DNA copies, by detecting a region in the EBER gene, using previously published primers [28] (Table 1). For this purpose, an EBV DNA (Advanced Biotechnologies) standard curve was prepared using reactions containing 2.4, 24, 240, 2400 or 24000 copies of EBV DNA per reaction. Each subject qPCR reaction mixture contained 22 ng subject DNA. Since 22 ng of DNA is the estimated genomic DNA content of 3.34 x 10^3^ cells and the viral load is reported as EBV DNA copies per 10^6^ PBMCs, we used the following formula for calculating the DNA load:

EBV DNA copies per 10^6^ PBMCs = (EBV DNA copy number per qPCR reaction x 10^6^) / (3.34 x 10^3^).

### Statistical analysis

A one-way ANOVA with a Tukey post-hoc test was used to compare means in mouse expression studies across groups with multiple treatments. Two-sample t-tests were performed to compare means in human studies of IL-17A and EBV DNA levels limited to two groups, the RA patients and the control group. Spearman’s rho coefficient and R^2^ were calculated to assess linear correlations between EBV copy numbers and the IL-17A levels in human samples. Statistical analysis was performed using PASW Statistics 18 for Windows. P-values <0.05 were considered statistically significant.

## Results

### EBV DNA modulates the expression of mouse Th17 and regulatory T cell activity markers

**Fig 1:**
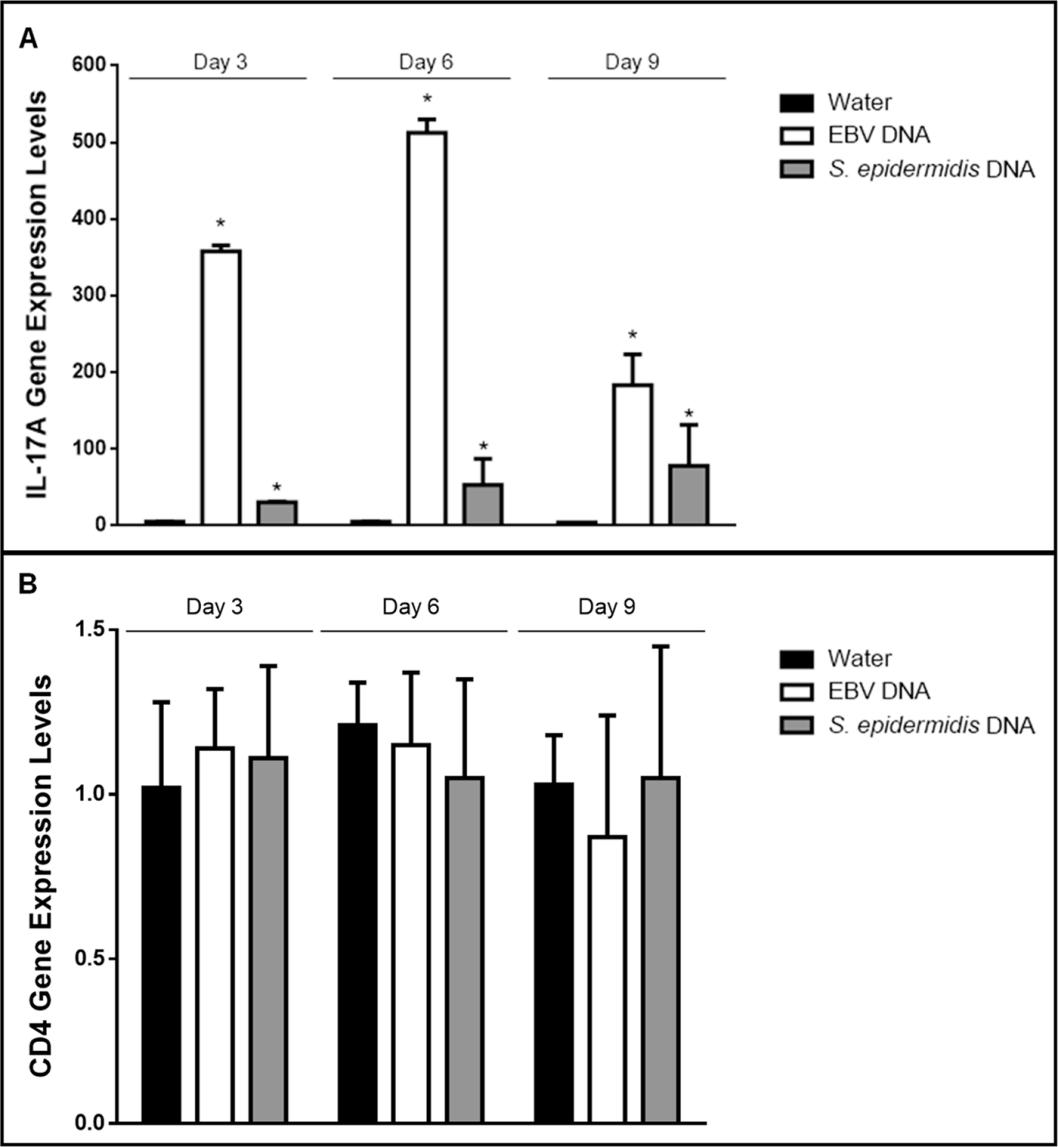
**Relative gene expression of (A) IL-17A and (B) CD4 in BALB/c mouse splenic tissues.** BALB/c mice were intraperitoneally injected with sterile distilled water, EBV DNA (144x10^3^ copies) or *S*. *epidermidis* DNA (28.3pg). Spleens were collected on days 3, 6 and 9 post-injection and relative gene expression was assessed by reverse transcriptase real-time PCR. Levels were normalized to those on day 3 post-injection with sterile distilled water. * indicates p<0.05 compared to water-injected group on respective day of injection.

**Fig 2:**
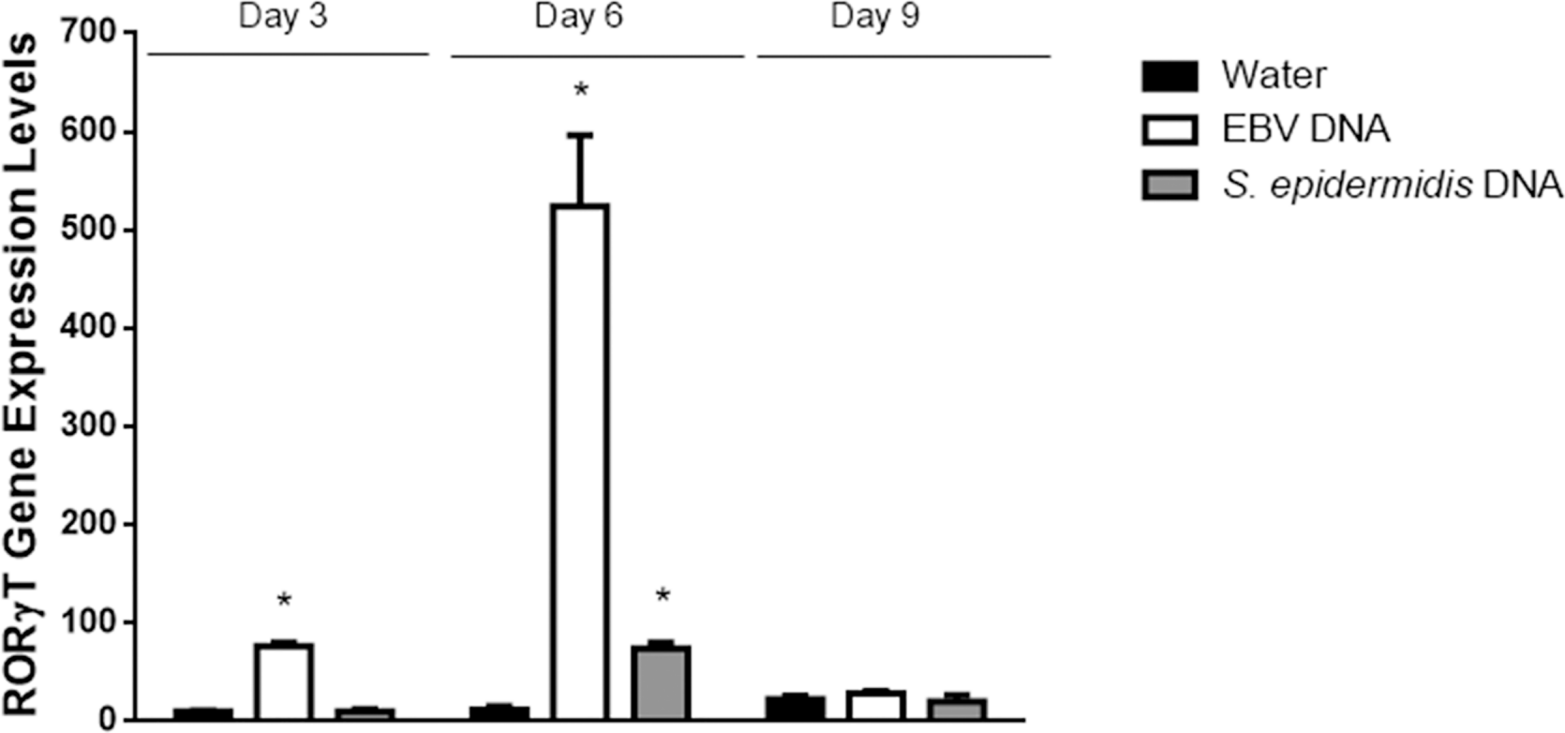
Relative gene expression of RORγT in BALB/c mouse splenic tissues. BALB/c mice were intraperitoneally injected with sterile distilled water, EBV DNA (144x10^3^ copies) or *S*. *epidermidis* DNA (28.3pg). Spleens were collected on days 3, 6 and 9 post-injection and RORγT relative gene expression was assessed by reverse transcriptase real-time PCR. Levels were normalized to those on day 3 post-injection with sterile distilled water. * indicates p<0.05 compared to water-injected group on respective day of injection.

Intraperitoneal injection of EBV DNA into BALB/c mice led to a 357-fold increase in the transcriptional levels of IL-17A in mouse splenic tissues on day 3 (p<0.001)), a 512-fold increase on day 6 (p<0.001) and a 183-fold increase on day 9 (p = 0.003) post-injection normalized to its expression on day 3 post-injection in the mouse group treated with sterile distilled water (Fig 1A). Although injection with *S*. *epidermidis* DNA resulted in some increase in IL-17A transcription, the highest fold increase was by 78 on day 9. This is consistent with our previous findings indicating that injecting mice with EBV DNA increases their serum IL-17A levels [13, 19, 29–31]. To examine whether this observation is due to augmented Th17 responses rather than an overall increase in the activity or numbers of CD4+ cells, we assessed CD4 gene expression levels in splenic tissues (Fig 1B). No significant changes were observed in the mRNA levels of this marker upon injection of EBV or *S*. *epidermidis* DNA on days 3, 6 or 9 post-injection. This rather indicates a particular increase in the Th17 response to EBV DNA. On the other hand, we detected a significant 76-fold increase in RORγT gene expression on day 3 post-injection (p<0.001) and a 524-fold increase on day 6 post-injection (p = 0.002) (Fig 2). The highest level of RORγT transcript detection upon treatment with *S*. *epidermidis* DNA was on day 6; this increase was by a 74-fold one. RORγT is a transcription factor that regulates the expression of IL-17A among other genes in Th17 cells [32] and that plays an essential role in Th17 cell differentiation [33, 34]. Hence, transcriptional level increases induced by *S*. *epidermidis* DNA in the expression of IL-17A and RORγT were notably less than those triggered by EBV DNA at all assessed time points.

**Fig 3:**
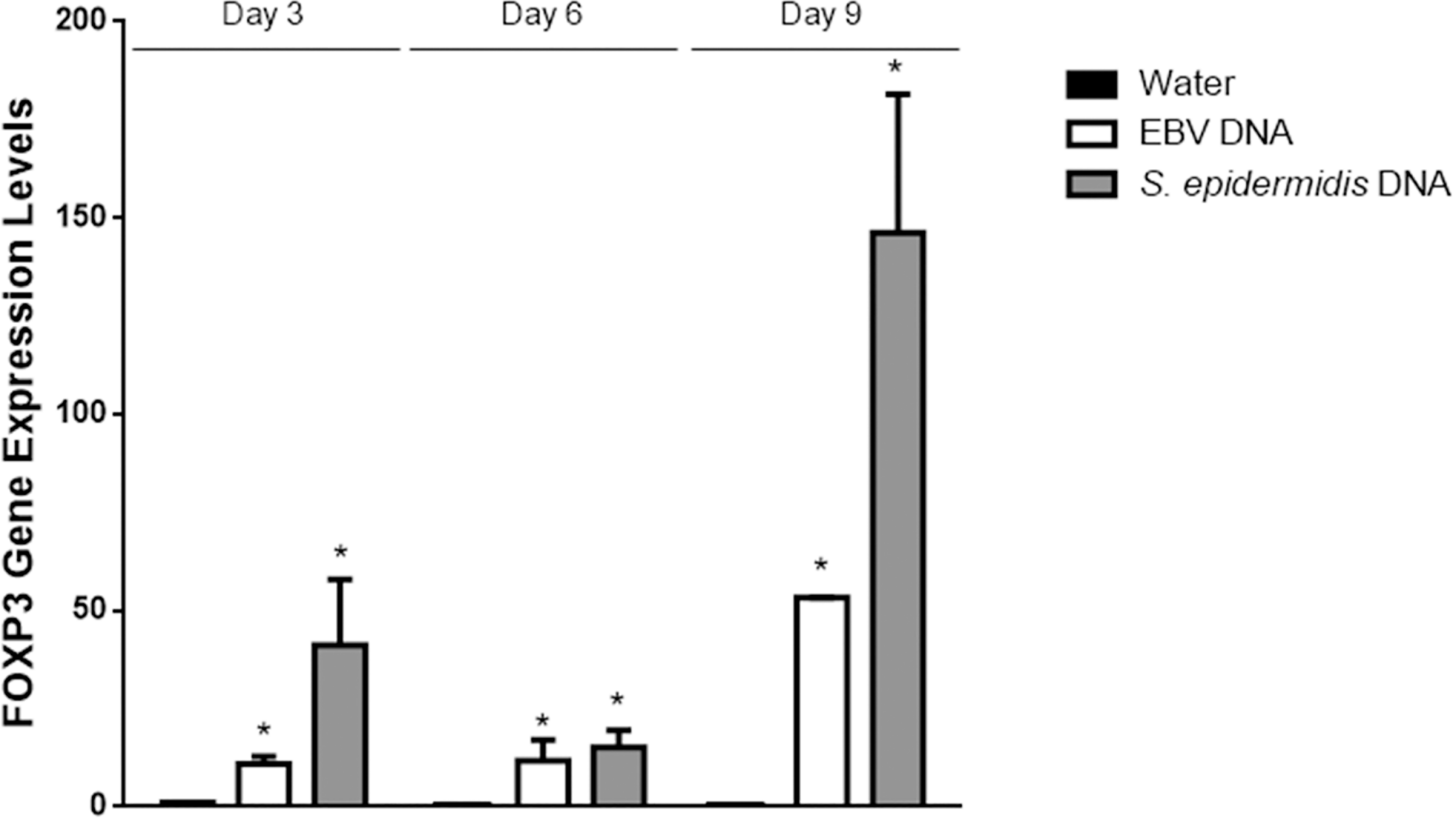
Relative gene expression of FOXP3 in BALB/c mouse splenic tissues. BALB/c mice were intraperitoneally injected with sterile distilled water, EBV DNA (144x10^3^ copies) or *S*. *epidermidis* DNA (28.3pg). Spleens were collected on days 3, 6 and 9 post-injection and FOXP3 relative gene expression was assessed by reverse transcriptase real-time PCR. Levels were normalized to those on day 3 post-injection with sterile distilled water. * indicates p<0.05 compared to water-injected group on respective day of injection.

**Fig 4:**
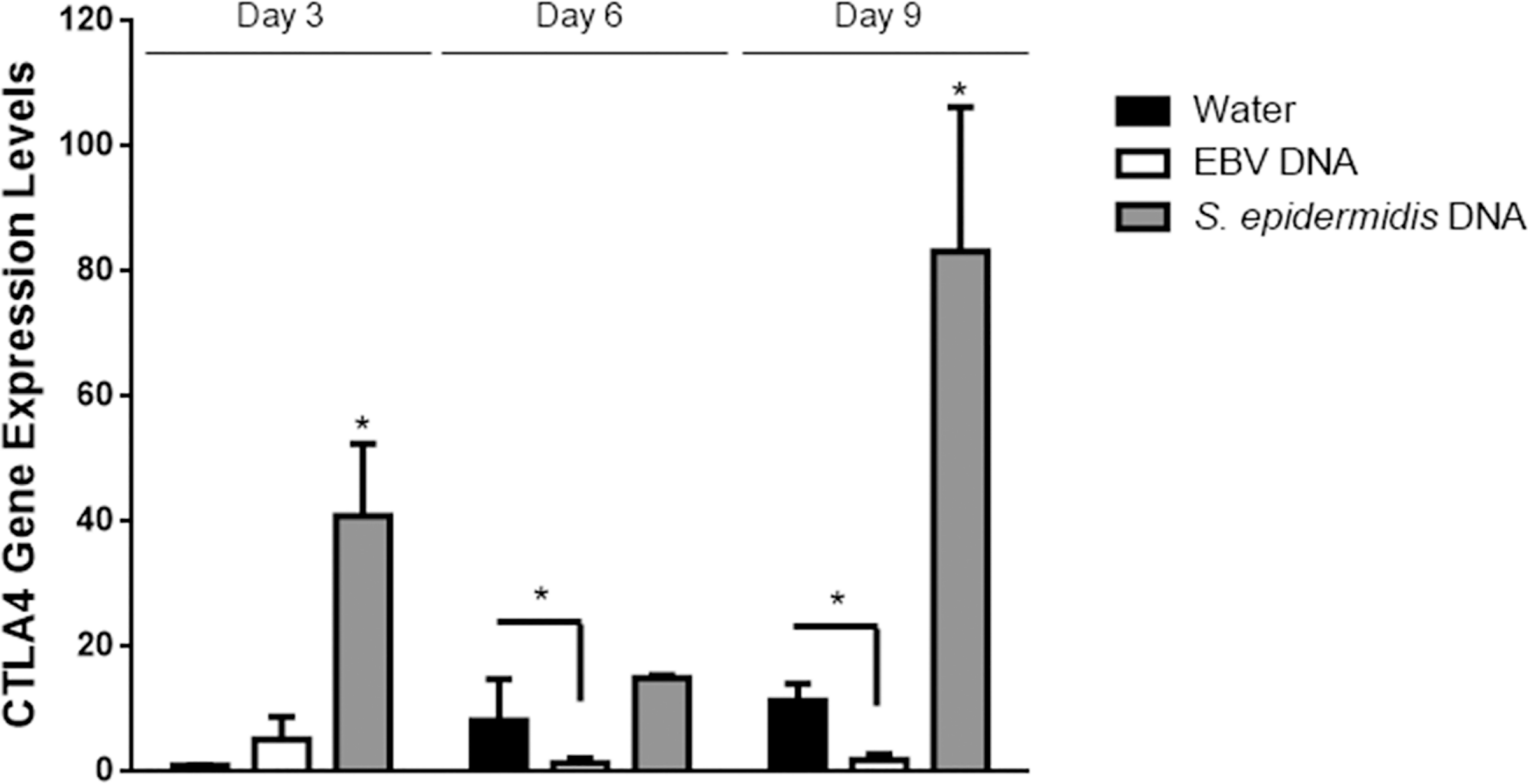
Relative gene expression of CTLA4 in BALB/c mouse splenic tissues. BALB/c mice were intraperitoneally injected with sterile distilled water, EBV DNA (144x10^3^ copies) or *S*. *epidermidis* DNA (28.3pg). Spleens were collected on days 3, 6 and 9 post-injection and CTLA4 relative gene expression was assessed by reverse transcriptase real-time PCR. Levels were normalized to those on day 3 post-injection with sterile distilled water. * indicates p<0.05 compared to water-injected group on respective day of injection.

To assess whether regulatory T cell capacity was also affected by mouse treatment with EBV DNA, we first assessed the expression of FOXP3 which is a key transcription factor required for the development of Tregs as well as their maintenance and function [35, 36]. Normalized to its expression in the mouse group treated with sterile distilled water on day 3 post-injection, the level of FOXP3 expression upon intraperitoneal injection of EBV DNA into BALB/c mice showed an 11-fold increase on day 3 (p<0.001), a 12-fold increase on day 6 (p = 0.005) and a 53-fold increase on day 9 (p = 0.005) post-injection (Fig 3). On the other hand, in contrast with the results observed for Th17 activity markers, the injection of *S*. *epidermidis* DNA also led to a 41-fold increase in FOXP3 gene expression on day 3 (p = 0.014), a 15-fold increase on day 6 (p = 0.009) and by a 146-fold increase on day 9 (p = 0.001) post-injection; these levels were thus higher than the increases observed upon injection with EBV DNA. In addition to assessing FOXP3, we examined the expression of CTLA4; this immunoregulatory marker is constitutively expressed in FOXP3+ Tregs in addition to non-Treg T lymphocyte populations following activation. CTLA4 is an inhibitory molecule related to the T cell costimulatory molecule CD28. CTLA4 and CD28 bind to shared ligands (CD80, CD86) on antigen presenting cells. While CD28 signaling promotes T cell activation, CTLA4 serves an immunoregulatory function, suppressing the T cell response [31]. Injecting EBV DNA did not lead to a statistically significant increase in the level of CTLA4 gene expression on any of the days tested (Fig 4). On the other hand, *S*. *epidermidis* DNA resulted in a significant 40-fold increase in the level of CTLA4 gene expression on day 3 (p<0.001), a 15-fold increase on day 6 (p = 0.001) and an 83-fold increase on day 9 (p = 0.026) post-injection normalized to its expression on day 3 post-injection with sterile distilled water. Notably, CTLA4 expression in the EBV DNA-injected group was not only lower than the *S*. *epidermidis* DNA-injected groups at all time points, but also significantly lower than the water injected group by about a 4-fold difference (p = 0.045) on day 6 and by a 6-fold difference (p = 0.041) on day 9 post-injection. This may indicate inhibitory effects of EBV DNA on the expression of CTLA4.

### Inhibition of TLR9 reduces IL-17A production in mouse peripheral blood mononuclear cells stimulated with EBV DNA

**Fig 5:**
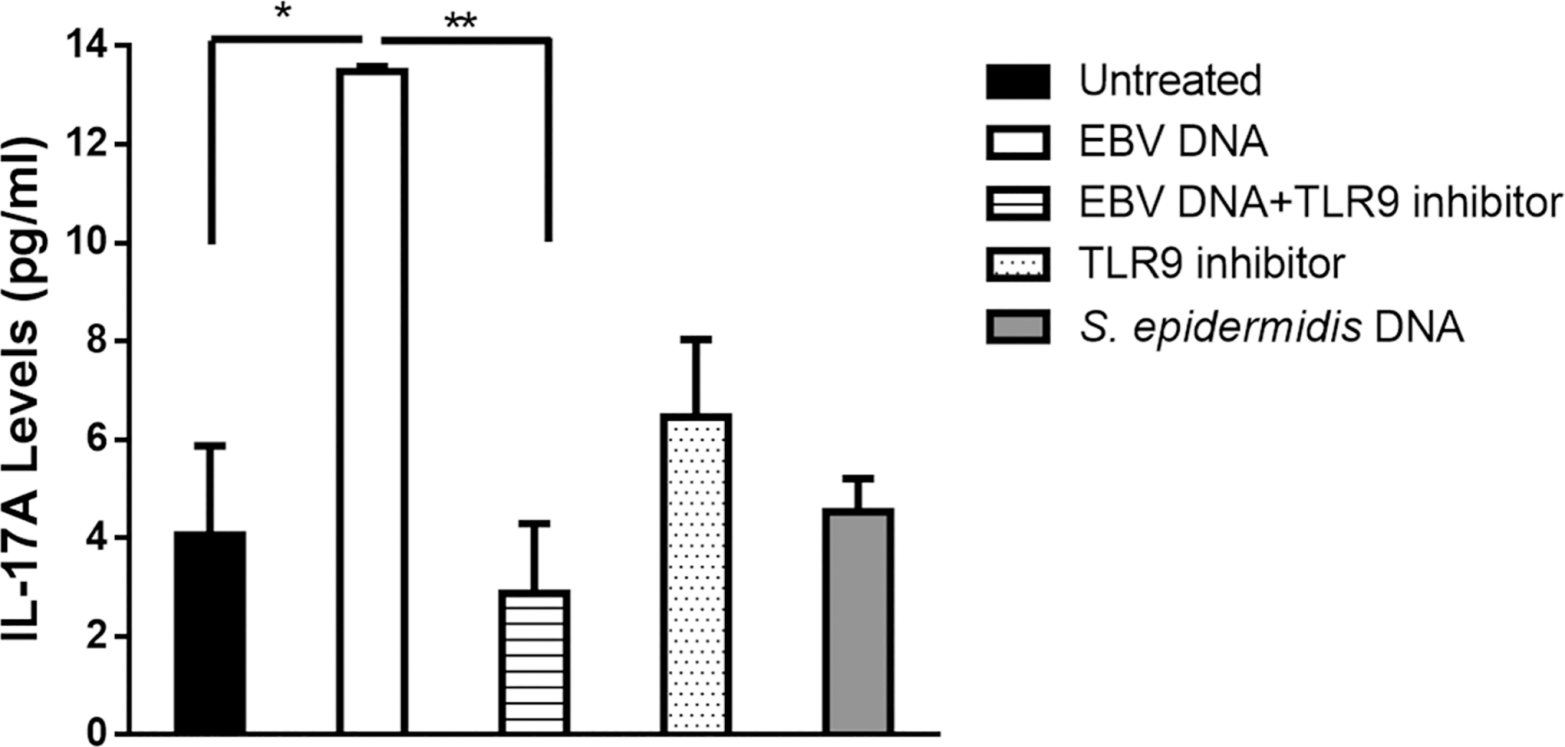
IL-17A levels from BALB/c mouse PBMC. PBMCs were cultured with EBV DNA (9000 copies), TLR9 inhibitor alone (1.4μM), EBV DNA and TLR9 inhibitor, or with *Staphyloccocus epidermidis* DNA. Untreated cells were examined as controls. After a 24-hour culture period IL-17A levels were assessed in the culture medium by ELISA. * indicates p<0.05 when compared to untreated cells; ** indicates p<0.05 when compared to cells cultured with EBV DNA.

To assess the mechanism by which EBV DNA triggers IL-17A expression in mice we examined the involvement of TLR9 in this pathway. CpG dinucleotides serve as a primary trigger for TLR9 when unmethylated and are present in this state within newly-formed EBV DNA in an infected cell and when a newly-formed virion is then released [22]. Therefore, we examined the response of mouse PBMCs to EBV DNA in the presence or absence of the TLR9 inhibitor ODN 2088. Incubation of mouse PBMCs with EBV DNA resulted in a 2.3-fold increase in the level of IL-17A production (Fig 5). On the other hand, culturing these cells with EBV DNA and the TLR9 inhibitor led to a decrease by 73.41% (p = 0.002) in IL-17A production compared to cells incubated with EBV DNA alone. Culturing cells with the TLR9 inhibitor alone did not result in significant changes. PBMCs cultured with *S*. *epidermidis* DNA, used as a non-viral DNA control, resulted in levels of IL-17A that were similar to those detected in untreated cells. This indicates that TLR9, in its capacity as an endosomal Pattern Recognition Receptor (PRR), is activated by DNA from EBV and results in enhanced production of IL-17A.

### The relationship between EBV DNA copy numbers and IL-17A levels has a propensity for linearity in rheumatoid arthritis subjects

Having observed that EBV DNA enhances IL-17A production in the murine system, we intended to examine whether similar effects for this viral DNA can be detected in humans in particular within the setting of an autoimmune disease. Therefore, we assessed both viral DNA copy numbers and IL-17A levels in both RA patients and matched controls.

**Fig 6:**
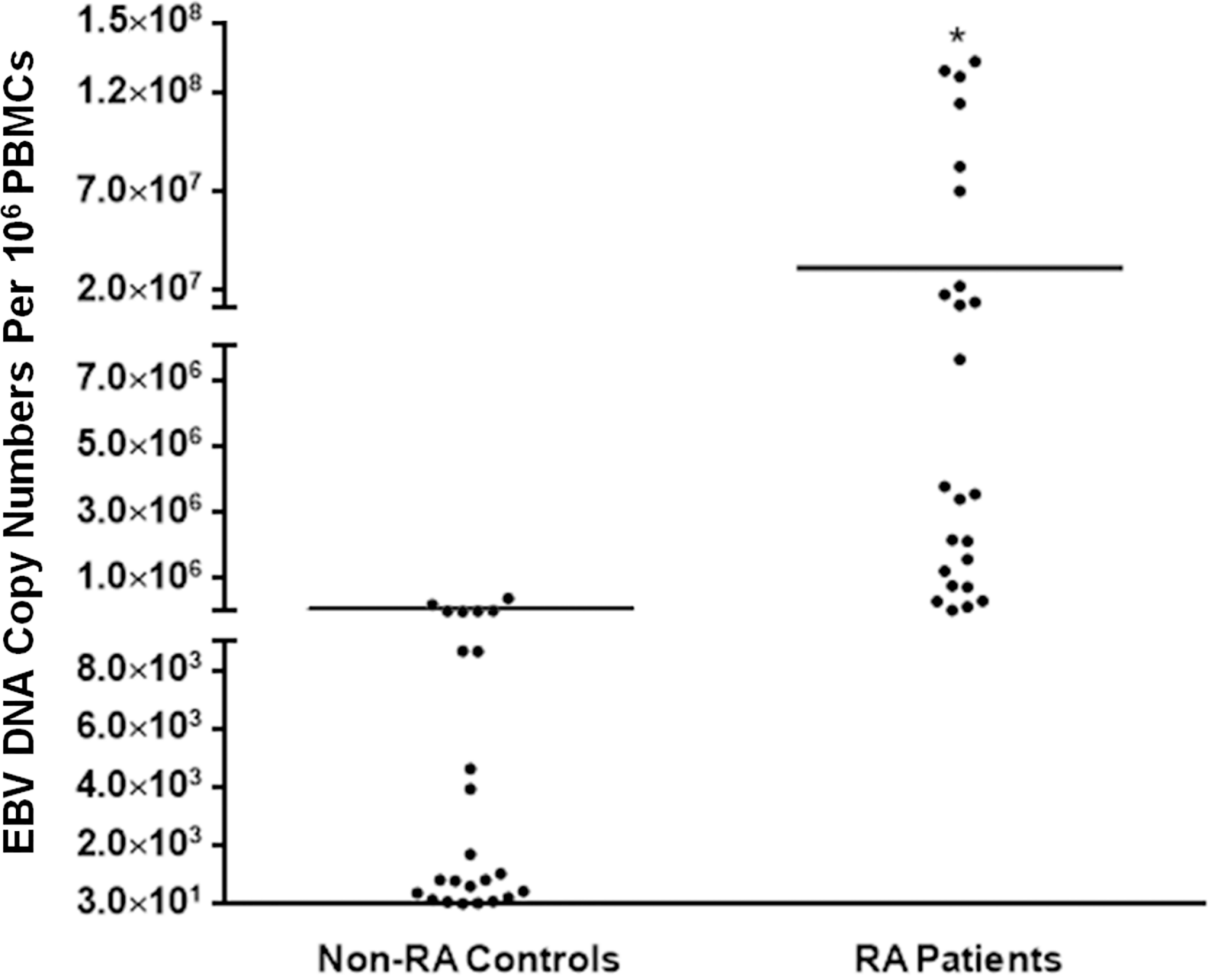
Average levels of EBV DNA in RA patients and non-RA controls. PBMCs were separated from blood collected from 24 RA patients and 24 non-RA controls. DNA was extracted from subject cells and then EBV DNA copy numbers were determined by qPCR. * indicates p<0.05 compared to controls.

**Fig 7:**
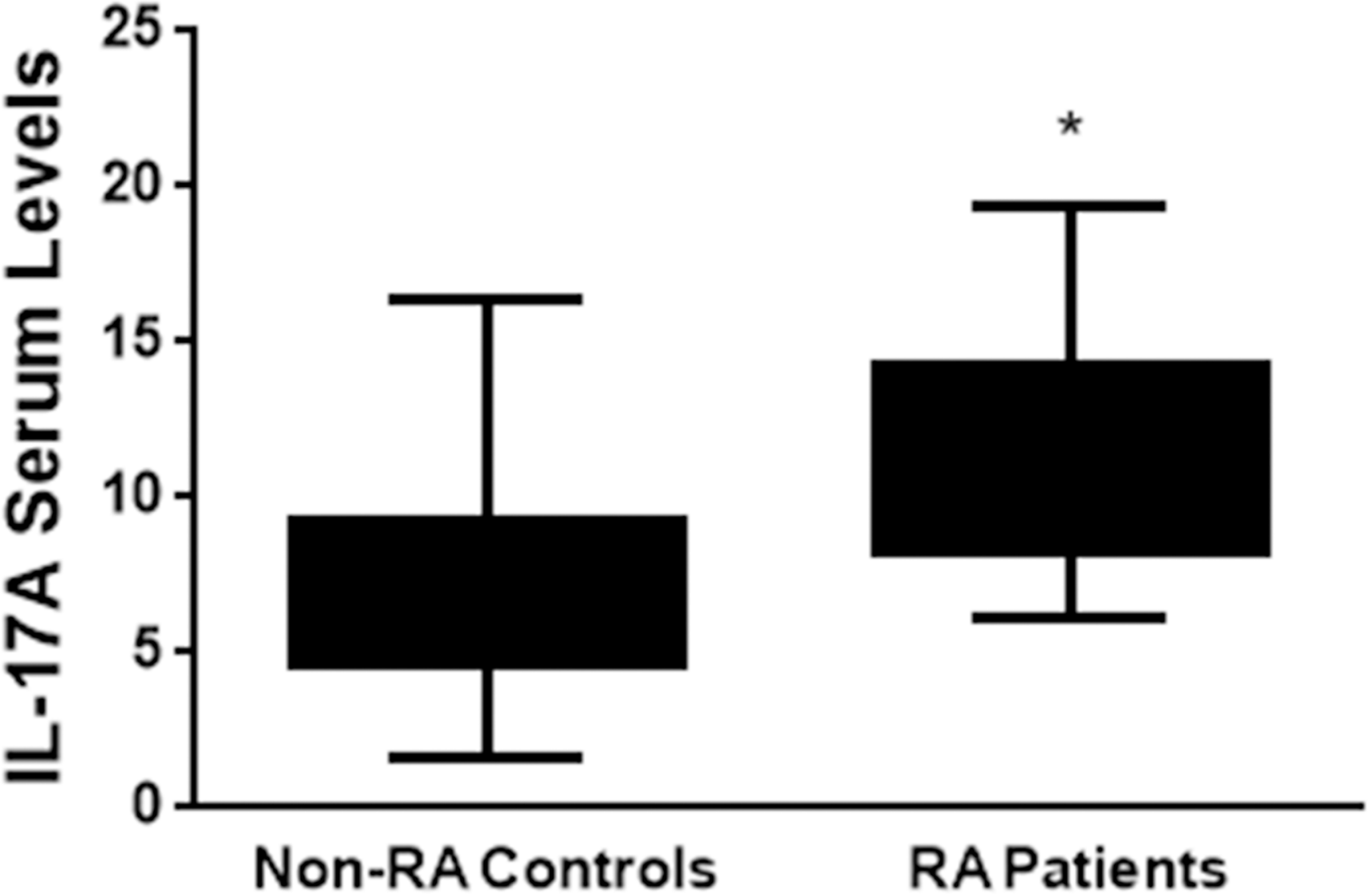
Average serum levels of IL-17A in RA patients and non-RA controls. Serum was collected from 24 RA patients and 24 non-RA controls. IL-17A levels were then assessed by ELISA. * indicates p<0.05 compared to controls.

**Fig 8:**
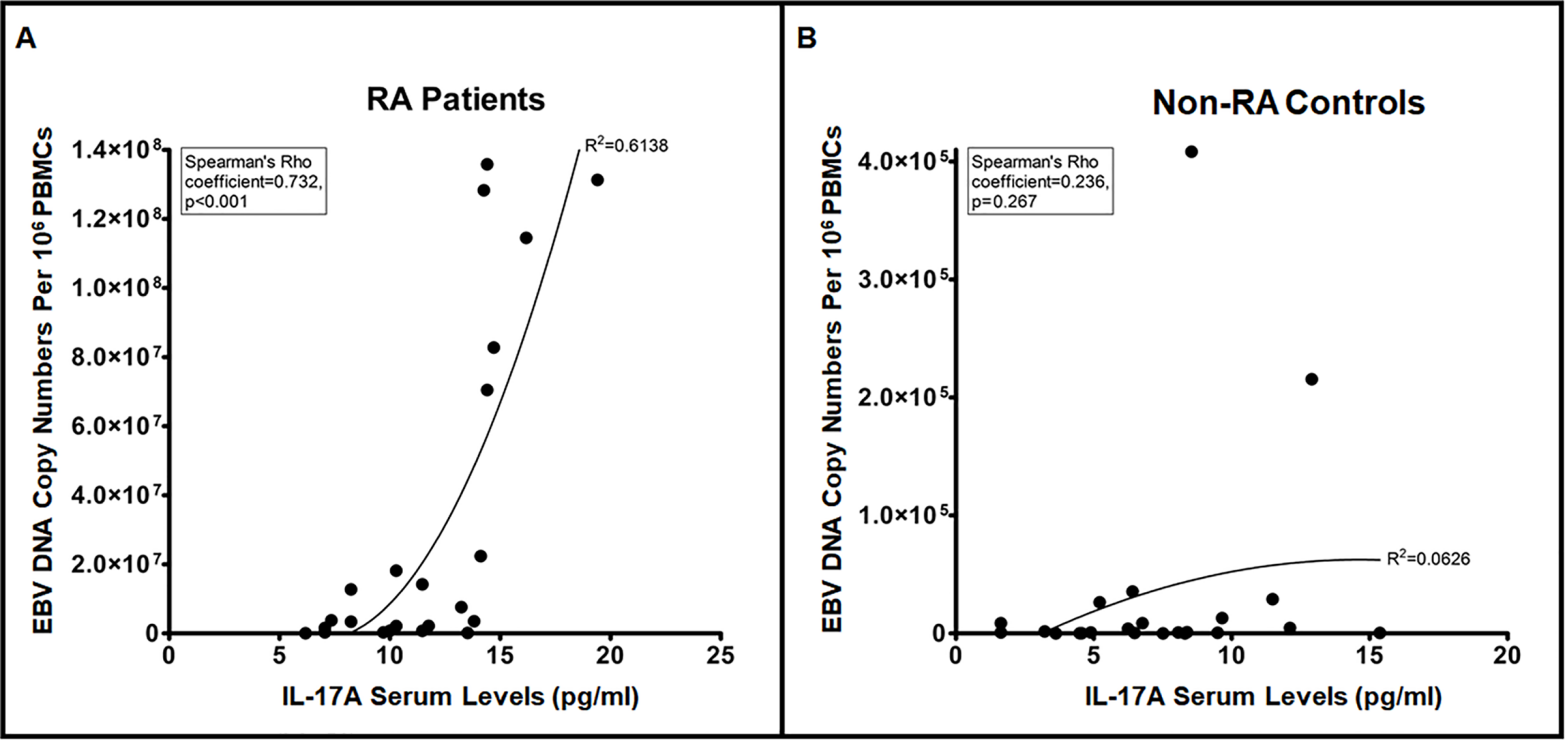
**Correlation between EBV DNA copy numbers and serum IL-17A levels in (A) RA patients and (B) non-RA controls.** EBV DNA copy numbers and IL-17A levels were assessed in RA and non-RA control subjects by qPCR and ELISA respectively.

Average EBV DNA copy numbers were significantly (p = 0.0026) higher in RA patients (31.61x10^6^) compared to controls (31.73x10^3^) (Fig 6). The RA patient group also had a significantly (p<0.001) higher IL-17A average level when compared to non-RA controls (Fig 7). While the average level in the RA patients group was 11.42 pg/ml, it was 7.3 pg/ml in the control group. Both of these findings were anticipated as our interest was in examining whether higher EBV DNA copy numbers resulted in proportionately higher serum IL-17A levels per individual. Therefore, the linearity of the relationship between EBV DNA copy numbers and serum IL-17A levels was evaluated. In RA patients (Fig 8A), the correlation between EBV DNA copy numbers and serum IL-17A levels indicated an R^2^ of 0.6138 and a Spearman’s Rho Coefficient of 0.732 (p<0.001) while the relationship between these two variables in the control group (Fig 8B) had an R^2^ of 0.0626 and a Spearman’s Rho Coefficient of 0.236 (p = 0.267). This indicates a statistically significant propensity for a linear relationship between serum IL-17A levels and EBV DNA copy numbers in RA patients but not in non-RA controls.

## Discussion

Induction of Th17-mediated immune responses and the activation of these cells has been consistently associated with the development of inflammation and autoimmunity [37]. In contrast, regulatory T cell functions have been considered principal among several complex regulatory mechanisms that sustain immune homeostasis, prevent autoimmune diseases, and control inflammation [38, 39]. In the study at hand, we observed that the transcriptional levels of RORγT, a transcription factor essential for the Th17 program, increased in mice splenic tissue upon intraperitoneal injection with EBV DNA. This is concurrent with our previous observations indicating that IL-17A increases at the protein level in mice injected with EBV DNA [13, 40]. On the other hand, transcriptional levels of assessed Th17 markers were lower in *S*. *epidermidis* DNA-injected mice than in those injected with EBV DNA on all of the examined days. This implies that EBV DNA has a higher potency of activating Th17 responses than *S*. *epidermidis* DNA.

In contrast to Th17 markers, those of regulatory T cells were higher in *S*. *epidermidis* DNA-injected mice than in EBV DNA-injected ones on all days tested. This may indicate the capability of the bacterial DNA to activate regulatory mechanisms in response to proinflammatory pathways that may have been triggered by the *S*. *epidermidis* DNA but not assessed in our current study. Transcriptional levels of FOXP3, a transcription factor essential for the Treg program, were induced by EBV DNA, however, to a lesser extent than the *S*. *epidermidis* DNA injection. A low level of FOXP3 transcriptional increase in the EBV DNA injected mice, by a 12-fold difference, coincided with the highest levels of Th17 markers on day 6 post-injection; this indicates that pro-Th17 responses likely affect regulatory T cell responses in an adverse manner. This notion is further supported by the detected transcriptional levels of CTLA4 which also were at their lowest on day 6 post-injection with EBV DNA. CTLA4 is a T cell regulatory activity marker and is constitutively expressed on Tregs. Not only were the levels of CTLA4 expression lower in the EBV DNA-injected group than the *S*. *epidermidis* DNA-injected group on all days tested, they were also significantly lower than the water-injected group on days 6 and 9 post-injection; the normalized level of CTLA4 was 4.39 in the water-injected group, while it was 1.23 in the EBV DNA-injected group (p = 0.0447) on day 6. On the other hand, its normalized level was 11.43 in the water-injected while it was 1.95 in the EBV DNA injected group (p = 0.0408) on day 9. This indicates that CTLA4 expression is rather suppressed by EBV DNA.

These results demonstrate that EBV DNA favors Th17 cell activity at the expense of regulatory T cells. This may be explained by the fact that critical factors required for Th17 lymphocytes, including the transcription factors STAT3 and RORγT, in addition to pro-inflammatory cytokines such as IL-6, IL-1β and IL-21 in the presence of TGF-β [41], simultaneously restrict the expression of factors needed for Treg programming such as STAT5 and FOXP3, which are usually induced by TGF-β alone [29, 42].

EBV DNA contains unmethylated CpG, the ligand and the activator of TLR9 [22]. Therefore, we assessed whether this receptor plays a role in EBV DNA-triggered IL-17A production. When mouse PBMCs were cultured with TLR9 inhibitor in presence of EBV DNA, there was a statistically significant decrease in IL-17A production. These observations indicate that TLR9 recognizes EBV DNA thus triggering this pathway. It has been previously reported that activation of monocytes by TLR9 agonists results in the secretion of TGFβ, IL-6, IL-1, IL-23 and IL-21. These factors may then promote IL-17A production [21].

Our observations regarding the effect of EBV DNA on IL-17A production have so far been in mice; therefore, we examined whether similar observations could be made in humans. Hence, we examined the effect of an EBV infection on IL-17A production in RA patients and controls. Our assumption was that levels of EBV DNA copies would correlate with IL-17A levels in assessed subjects. As expected, the average serum IL-17A levels are higher in RA patients than non-RA subjects. This is similar to what other studies have observed [43, 44]. In addition, we have shown that the average EBV DNA level are higher in RA patients than non-RA subjects. Other studies have also reported an increased EBV DNA load in RA patients compared to control subjects [45–48]. Our analysis indicates that in RA patients, a propensity for a linear correlation exists between EBV DNA levels and serum IL-17A; however, such a linearity was not detectable in non-RA controls. This may indicate that RA patients become more prone to IL-17A production in the presence of EBV DNA due to preexistent rather over-active pro-inflammatory pathways. Alternatively, this may indicate the presence of a particular genetic factor in RA patients that makes them more prone to IL-17A production due to EBV DNA. Whether reactivation of EBV replication would result in a flare up or enhanced severity of disease remains to be examined. Any association between disease status and EBV DNA copy numbers will be considered in future studies.

In conclusion, our data indicates the involvement of EBV DNA in inflammatory reactions via the modulation of two major cell compartments with relatively opposing functions; on one hand, inducing Th17 cells which contribute to the development of inflammation and autoimmunity by producing IL-17A, and on the other hand, repressing regulatory T cell activities which are responsible for suppressing inflammation and restricting the development of autoimmunity. This enhancement of IL-17A appears to be mediated via TLR9. This may indicate a possible utility for TLR9 inhibitors as therapeutic agents in subjects with an autoimmune disease, such as RA. For example, IRS 954 (a TLR7/TLR9 inhibitor) has been reported to have a potential in reducing pathogenesis of autoantibody production and autoimmune tissue injury in SLE, another autoimmune disease associated with EBV [30]. IRS 954 is still in its pre-clinical trial phase. Whether other oligodeoxynucleotides, such as IMO-3100, another TLR7/TLR9 antagonist in Phase II of clinical trials, may be useful remains to be seen [49].
